# The increasing threat to stratospheric ozone from dichloromethane

**DOI:** 10.1038/ncomms15962

**Published:** 2017-06-27

**Authors:** Ryan Hossaini, Martyn P. Chipperfield, Stephen A. Montzka, Amber A. Leeson, Sandip S. Dhomse, John A. Pyle

**Affiliations:** 1 Lancaster Environment Centre, Lancaster University, Lancaster LA1 4YQ, UK; 2 School of Earth and Environment, University of Leeds, Leeds LS2 9JT, UK; 3 National Centre for Earth Observation, University of Leeds, Leeds LS2 9JT, UK; 4 National Oceanic and Atmospheric Administration, Boulder, Colorado 80305, USA; 5 Department of Chemistry, University of Cambridge, Cambridge CB2 1EW, UK; 6 National Centre for Atmospheric Science, University of Cambridge, Cambridge CB2 1EW, UK

## Abstract

It is well established that anthropogenic chlorine-containing chemicals contribute to ozone layer depletion. The successful implementation of the Montreal Protocol has led to reductions in the atmospheric concentration of many ozone-depleting gases, such as chlorofluorocarbons. As a consequence, stratospheric chlorine levels are declining and ozone is projected to return to levels observed pre-1980 later this century. However, recent observations show the atmospheric concentration of dichloromethane—an ozone-depleting gas not controlled by the Montreal Protocol—is increasing rapidly. Using atmospheric model simulations, we show that although currently modest, the impact of dichloromethane on ozone has increased markedly in recent years and if these increases continue into the future, the return of Antarctic ozone to pre-1980 levels could be substantially delayed. Sustained growth in dichloromethane would therefore offset some of the gains achieved by the Montreal Protocol, further delaying recovery of Earth’s ozone layer.

## Abstract (web summary)

Chlorine-containing species deplete stratospheric ozone and while chlorofluorocarbons have been drastically reduced, dichloromethane concentrations have recently increased rapidly. Hossaini *et al*. show that continued growth at this rate could result in important delays to Antarctic ozone recovery.

In the 1970s, it was recognized that chlorine and bromine released from long-lived anthropogenic compounds, such as chlorofluorocarbons (CFCs) and halons, could destroy ozone in the stratosphere^1^,^2^. Industrial emissions of these halocarbons have led to widespread depletion of Earth’s ozone layer in recent decades, including the Antarctic ‘Ozone Hole’ phenomenon^3^,^4^,^5^. Peak ozone depletion was observed around the turn of the century, when globally the stratospheric ozone column was reduced by ∼5% relative to 1980 levels (a benchmark before which substantial ozone depletion had not been observed). While ozone depletion today remains a persistent environmental issue, there are signs that ozone recovery is underway^6^,^7^,^8^,^9^, owing to controls on the production of ozone-depleting compounds introduced by the 1987 Montreal Protocol and its amendments^10^,^11^. Given compliance with the Protocol, stratospheric levels of chlorine derived from controlled ozone-depleting compounds should continue to steadily decline in coming decades. As a result, stratospheric column ozone is projected to return to pre-1980 levels in the middle to latter half of this century, depending on location^7^,^12^.

Several human-produced chlorocarbons not controlled by the Montreal Protocol are present in Earth’s atmosphere. Among the most abundant of these compounds is dichloromethane (CH_2_Cl_2_)—an industrial solvent also used as a feedstock in the production of other chemicals, among other applications^13^,^14^. Unlike CFCs, which are virtually inert in the troposphere and have long atmospheric lifetimes (decades to centuries), CH_2_Cl_2_ is a so-called very short-lived substance (VSLS)^15^. Historically, VSLS have been thought to play a minor role in stratospheric ozone depletion due to their relatively short atmospheric lifetimes (typically <6 months) and therefore low atmospheric concentrations. However, substantial levels of both natural and anthropogenic VSLS have been detected in the lower stratosphere^15^,^16^,^17^,^18^ and numerical model simulations suggest a significant contribution of VSLS to ozone loss in this region^19^,^20^,^21^. Long-term measurements of CH_2_Cl_2_ reveal that its tropospheric abundance has increased rapidly in recent years^15^,^21^,^22^,^23^. For example, between 2000 and 2012, surface concentrations of CH_2_Cl_2_ increased at a global mean rate of almost 8% per year, with the largest growth observed in the Northern Hemisphere (NH)^21^. Given that natural emissions of CH_2_Cl_2_ are small, this recent growth likely reflects an increase in industrial emissions^15^. While the precise nature of the source remains poorly characterized, industrial CH_2_Cl_2_ emissions from Asia—in particular from the Indian subcontinent—appear to be growing in importance^23^. The impact of these observed changes and continued CH_2_Cl_2_ growth in coming decades on the timescale of stratospheric ozone recovery have not yet been considered.

Here, a state-of-the-art global chemical transport model (CTM) is used to examine the sensitivity of future stratospheric chlorine and ozone levels to sustained CH_2_Cl_2_ growth. CTMs are commonly used to investigate detailed chemical processes and here, because transport is specified in the model, we are able to isolate the ozone response solely to increasing levels of CH_2_Cl_2_ in the atmosphere. We find that continued growth in the atmospheric loading of CH_2_Cl_2_ could offset some of the future benefits of the Montreal Protocol and lead to a substantial delay (more than a decade) in the recovery of stratospheric ozone over Antarctica.

## Results

### Recent CH_2_Cl_2_ trends and future growth scenarios

**Figure 1:**
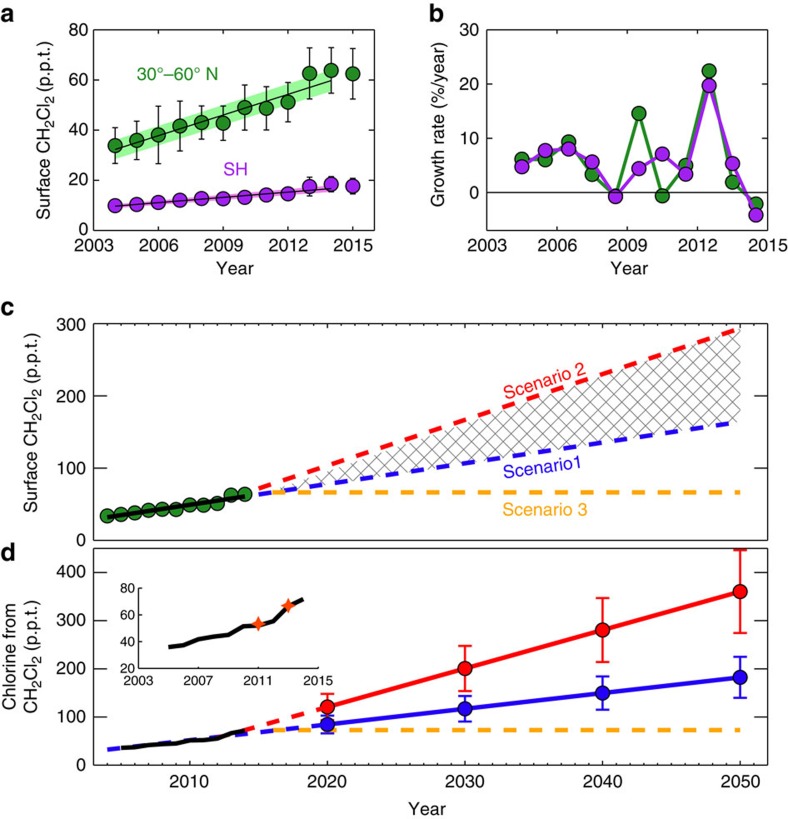
Observed trends and growth rate of surface CH_2_Cl_2_ and simulated stratospheric loading of chlorine. (**a**) CH_2_Cl_2_ surface mixing ratio in p.p.t. from 2004 to 2015 derived from NOAA measurements as the annual mean observed at 4 sites in the SH, and 5 sites in the NH between 30° and 60° N (ref. 36). The time series is an update of ref. 21, years 2014 and 2015 are new data. Error bars denote ±1 s.d. and the solid lines denote a linear fit to these data with the shaded regions representing ±1 s.d. uncertainty on the fit. (**b**) Corresponding CH_2_Cl_2_ growth rates (% per year). (**c**) Observed surface CH_2_Cl_2_ mixing ratio in the NH (green circles, as in **a**) and trend (black line), along with projections of surface CH_2_Cl_2_ between 30 and 60° N latitude under future scenarios (dashed lines); CH_2_Cl_2_ increases at the mean rate observed over the 2004–2014 period (Scenario 1, blue), CH_2_Cl_2_ increases at the mean rate observed over the 2012–2014 period (Scenario 2, red) and CH_2_Cl_2_ remains at 2016 levels (Scenario 3, no future growth, orange). (**d**) Modelled chlorine (p.p.t.) from CH_2_Cl_2_ entering the stratosphere in the recent past and projections. This is derived by multiplying the simulated CH_2_Cl_2_ mixing ratio at the tropical tropopause by 2, to account for the 2 Cl atoms in the molecule. Data between 2005 and 2013 are an update of ref. 22, while subsequent years and future projections are from this study. Annual means in decadal intervals (2020–2050) are shown (filled circles) with ±1 s.d. (error bars) for Scenarios 1 (blue) and 2 (red). Solid lines denote a linear fit to these data, dashed portions extrapolate this fit prior to 2020. The orange line (dashed throughout) represents Scenario 3 (no future growth). Inset; Enlarged model curve for 2004–2014 with observed estimates from NASA aircraft measurements (stars).

Figure 1 shows the measured surface abundance of CH_2_Cl_2_ from the National Oceanic and Atmospheric Administration (NOAA) long-term surface monitoring network. Globally, CH_2_Cl_2_ concentrations approximately doubled between 2004 and 2014 (Fig. 1a), although growth rates varied considerably during this period (Fig. 1b). The abundance of CH_2_Cl_2_ at mid-latitudes in the NH is around a factor of 3 greater than in the Southern Hemisphere (SH), reflecting NH industrial sources. At present, it is unknown if a single industrial application of CH_2_Cl_2_, or several, is contributing to the observed upward trend. As a common solvent, CH_2_Cl_2_ has numerous applications, which include use in metal cleaning/degreasing, in paint remover, and use by the pharmaceutical industry for preparing drugs. It is also used as blowing agent in production of foam plastics. A specific use of CH_2_Cl_2_, which seems likely to have increased in recent years, is in the manufacture of hydrofluorocarbons—the non-ozone-depleting chemicals used as replacements for CFCs and hydrochlorofluorocarbons (HCFCs). Given these sources, it is probable that demand for CH_2_Cl_2_ from developing countries now, and in coming years, will be relatively high. This is supported by elevated levels of CH_2_Cl_2_ detected over Asia, where Indian emissions are estimated to have increased by two- to fourfold between 1998 and 2008 (ref. 23).

**Figure 2:**
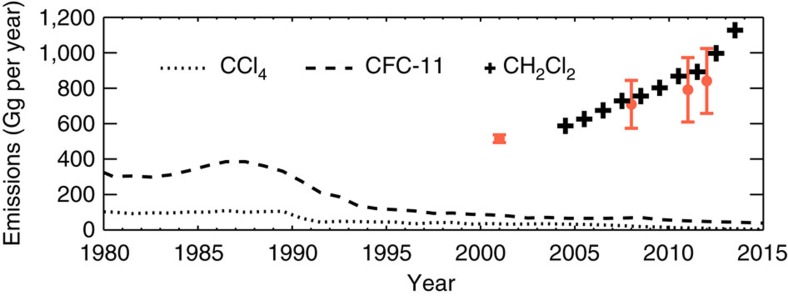
Time trend in global halocarbon emissions. Emissions derived from a simple 1-box model for CCl_4_ (dotted line), CFC-11 (dashed line) and CH_2_Cl_2_ (crosses) in units of Gigagrams (Gg) of source gas per year. Calculation for CH_2_Cl_2_ based on a parameterized global mean lifetime of 0.43 years. Also shown are recent independent estimates of CH_2_Cl_2_ emissions (orange points) from the AGAGE 12-box model^15^. Error bars denote uncertainty range.

Based on the NOAA surface measurements presented in Fig. 1a, we estimate a global emission source of around 1 Tg CH_2_Cl_2_ per year to sustain the observed CH_2_Cl_2_ concentrations in recent years (Fig. 2). We note that this is a far larger source than that of individual CFCs and other long-lived ozone-depleting gases (for example, carbon tetrachloride) in the 1980s, when emissions of those gases peaked. For CH_2_Cl_2_, and other VSLS more generally, relatively large emissions do not have the same impact on atmospheric concentrations, compared to say CFCs, as CH_2_Cl_2_ is more rapidly oxidized in the troposphere and has a much shorter atmospheric lifetime.

Two future scenarios encompassing potential surface CH_2_Cl_2_ increases from 2015 to 2100 have been derived and are considered in our forward model simulations. Both are based on observed long-term surface trends (Fig. 1c), and are designed to test the sensitivity of ozone to potential future changes in chlorine derived from CH_2_Cl_2_ growth. Scenario 1 assumes that surface CH_2_Cl_2_ continues to increase at the mean rate observed during the 2004–2014 period: 2.85 parts per trillion (p.p.t.) per year at mid-latitudes in the NH. Scenario 2, a more extreme growth scenario to test the sensitivity of ozone to larger CH_2_Cl_2_ increases, assumes CH_2_Cl_2_ continues to increase at the mean rate observed in the 2012–2014 period only: 6.1 p.p.t. per year. This period saw comparatively large CH_2_Cl_2_ growth compared to other recent years (Fig. 1b). In addition to the two growth scenarios, we also consider a third scenario in which no further CH_2_Cl_2_ growth occurs post 2016 (Methods section). In this scenario (Scenario 3), surface CH_2_Cl_2_ concentrations are fixed at 2016 levels throughout the forward simulation.

Constrained by the growth scenarios, our model simulations show a monotonic increase in chlorine from CH_2_Cl_2_ entering the stratosphere (Fig. 1d) in coming decades, from ∼70 p.p.t. Cl in 2014, to ∼180 p.p.t. Cl or ∼360 p.p.t. Cl by 2050, under Scenarios 1 or 2, respectively. Critically, the model reproduces well observed levels of CH_2_Cl_2_ around the tropopause in the recent past (Fig. 1d, inset) and, therefore, the stratospheric chlorine perturbation in response to increasing surface CH_2_Cl_2_ concentrations is realistic in our simulations.

### Impact of CH_2_Cl_2_ growth on stratospheric inorganic chlorine

**Figure 3:**
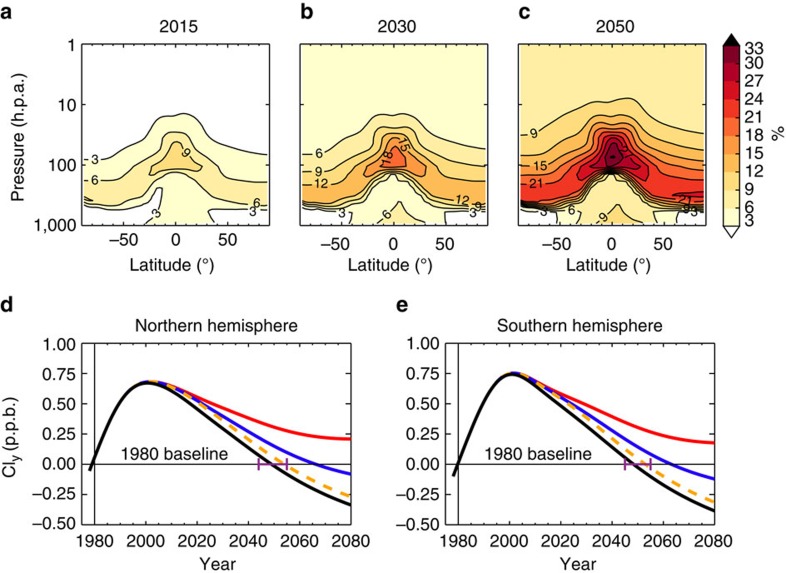
Contribution of CH_2_Cl_2_ to stratospheric inorganic chlorine and changes in total inorganic chlorine relative to 1980 baseline. (**a**–**c**) Percentage (%) of total stratospheric inorganic chlorine (Cl_y_) derived from CH_2_Cl_2_ in 2015, 2030 and 2050 under model run Scenario 1 (assuming CH_2_Cl_2_ continues to increase at the mean rate observed over the 2004–2014 period). (**d**,**e**) Modelled annual mean mid-latitude Cl_y_ change in northern (35–60° N) and southern (35–60° S) hemisphere. The Cl_y_ change is expressed in parts per billion (p.p.b.) relative to 1980 baseline at 50 hPa (lower stratosphere). Cl_y_ changes are shown for model simulations without CH_2_Cl_2_ (black) and with CH_2_Cl_2_ under growth Scenarios 1 (blue, see above) and 2 (red, assuming CH_2_Cl_2_ increases at the mean rate observed over the 2012–2014 period), and for the no additional growth Scenario 3 (orange). The projected dates when Cl_y_ returns to 1980 levels in the NH are 2050 (no CH_2_Cl_2_), 2067 (Scenario 1) and 2054 (Scenario 3). In the SH: 2049 (no CH_2_Cl_2_), 2064 (Scenario 1) and 2053 (Scenario 3). The horizontal purple lines show best estimated range of 1980 return dates from previous CCMs^6^ which did not include CH_2_Cl_2_.

The dissociation of ozone-depleting compounds in the stratosphere liberates chlorine radicals which catalyse ozone loss. Owing to its relatively short stratospheric partial lifetime (of the order of 1–2 years in our model outside of the poles), CH_2_Cl_2_ dissociates rapidly and thereby makes its largest relative contribution to the pool of inorganic chlorine (Cl_y_) in the lowermost stratosphere, at low latitudes (Fig. 3a–c). At present, CH_2_Cl_2_ accounts for <10% of stratospheric Cl_y_, although this contribution would increase significantly in coming decades if CH_2_Cl_2_ growth continues and as chlorine from long-lived gases decreases. By 2050 under Scenario 1, CH_2_Cl_2_ is projected to account for 20–30% of total Cl_y_ in the lower stratosphere. An examination of the stratospheric Cl_y_ trend in recent decades in our model reveals a peak in Cl_y_ around the turn of the century at mid-latitudes (Fig. 3d,e). In the absence of CH_2_Cl_2_, here lower stratospheric Cl_y_ is projected to return to pre-1980 levels by 2049, in line with ongoing decreases in levels of CFCs and other controlled long-lived Cl_y_ precursors^7^. When CH_2_Cl_2_ growth is considered, this Cl_y_ return date is delayed by around 15–17 years under Scenario 1. Under Scenario 2—an extreme scenario—the Cl_y_ return date occurs after 2080.

The delay in the Cl_y_ return date discussed above under Scenario 1 is significant and is additional to, and of a similar magnitude, to other factors which have previously been considered when assessing the uncertainty in return dates, for example, coupled chemistry-transport differences between climate models or different future greenhouse gas scenarios. The effect on the Cl_y_ return date is also much larger than the influence of potentially eliminating remaining small levels of production or emission of CFCs and HCFCs^24^. In the upper stratosphere, Cl_y_ return dates occur later than in the lower stratosphere and are less sensitive to future CH_2_Cl_2_ growth (Supplementary Fig. 1). Here, the contribution of CH_2_Cl_2_ to total Cl_y_ remains at <10% in 2050 (Fig. 3c).

### Impact of past and potential future CH_2_Cl_2_ growth on ozone

We consider next the impact of CH_2_Cl_2_ on ozone. Ozone is most sensitive to CH_2_Cl_2_ in polar regions; by the time air reaches high latitudes all chlorine that entered the stratosphere as CH_2_Cl_2_ has been converted to Cl_y_. The largest ozone decreases attributable to CH_2_Cl_2_ are simulated in the SH, where the Antarctic Ozone Hole—the most drastic manifestation of the effect of halogen-driven ozone loss—forms each spring. Although modest, the impact of CH_2_Cl_2_ is non-negligible in the present day with springtime column ozone up to ∼3%, or 6 dobson units (DU), lower in simulations in which CH_2_Cl_2_ is considered relative to an atmosphere without CH_2_Cl_2_ in 2016 (Supplementary Fig. 2). The equivalent relative ozone decrease in the spring of 2010 was ∼1.5% (3 DU), which allows quantification of CH_2_Cl_2_ increases on polar ozone depletion during spring, over this period, and highlights that this impact has already doubled in the past 6 years alone.

**Figure 4:**
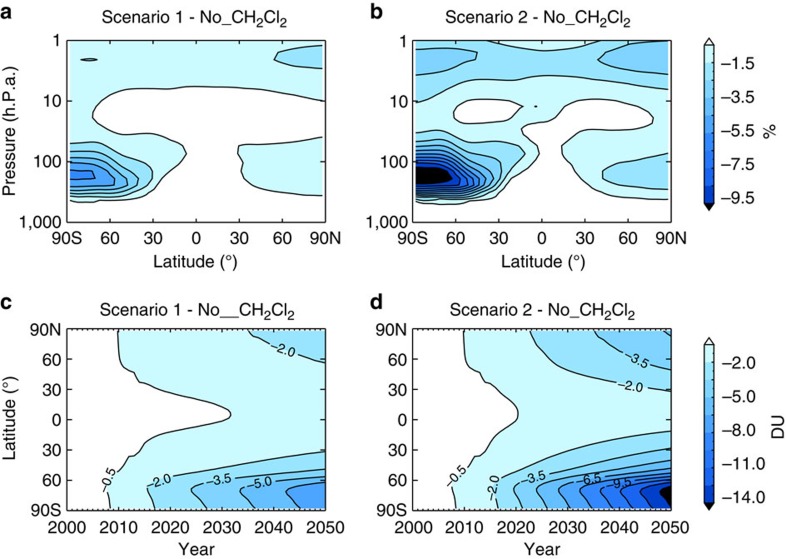
Stratospheric ozone decreases due to CH_2_Cl_2_. (**a**) Difference in zonal mean annual mean ozone (%) between run Scenario 1, assuming surface CH_2_Cl_2_ continues to increase at the mean rate observed over the 2004–2014 period, and run no_CH_2_Cl_2_ in 2050. (**b**) As **a** for Scenario 2, assuming CH_2_Cl_2_ continues to increase at the mean rate observed over the 2012–2014 period. (**c**) Difference in zonal mean annual mean column ozone (DU) between run Scenario 1 and run no_CH_2_Cl_2_ as a function of year. (**d**) As **c** for Scenario 2.

Figure 4 shows simulated ozone changes due to CH_2_Cl_2_ in the recent past and future period. Ozone loss due to further CH_2_Cl_2_ growth is projected to increase significantly in coming decades. By 2050, expressed as an annual mean, ozone is ∼6% lower in the Antarctic lower stratosphere under growth Scenario 1 (Fig. 4a), and annual mean column ozone is decreased by up to 8 DU, relative to the no CH_2_Cl_2_ simulation (Fig. 4c), against a background of recovering ozone. At mid-latitudes, column ozone decreases are smaller (up to several DU) and despite CH_2_Cl_2_ making a relatively large contribution to Cl_y_ in the tropics, ozone decreases here remain small (<1%). Recall, our simulations in the future period reflect the ozone response to changes in projected stratospheric composition only, comparing scenarios with CH_2_Cl_2_ growth to one with no CH_2_Cl_2_. Future ozone is also expected to be influenced by other factors, including climate-driven changes to stratospheric temperature and circulation^7^,^12^,^25^ and possibly due to changes in natural halocarbon emissions^26^. By isolating the ozone response due to CH_2_Cl_2_, we highlight its increasing influence on ozone evolution in coming decades. Such findings could also be relevant from a climate perspective^21^ as ozone absorbs both ultraviolet and infrared radiation, and as ozone perturbations in the lower stratosphere cause a relatively large radiative effect^27^.

**Figure 5:**
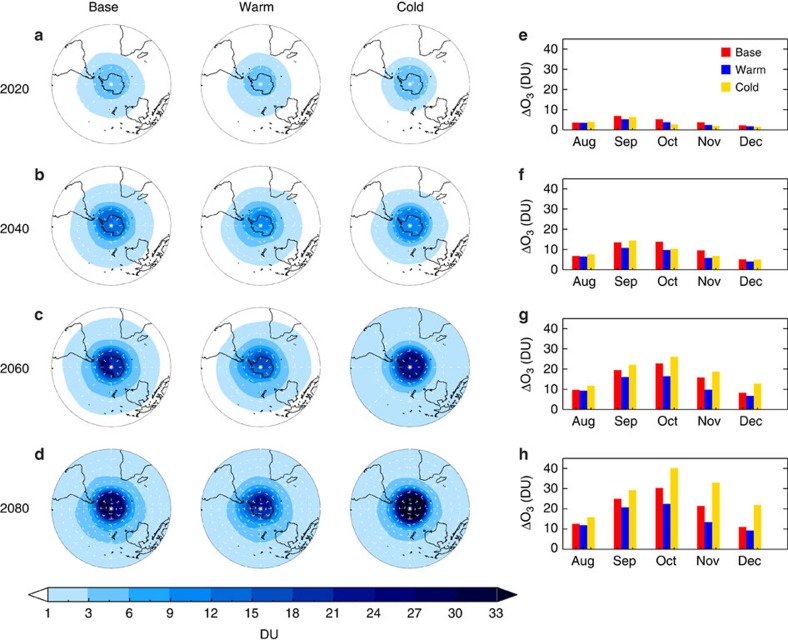
Sensitivity of SH ozone loss due to CH_2_Cl_2_ to different stratospheric meteorology. (**a**–**d**) Spring-time mean column ozone decrease (DU) in SLIMCAT in 2020, 2040, 2060 and 2080 calculated as run no_CH_2_Cl_2_ minus run Scenario 1 (assuming surface CH_2_Cl_2_ continues to increase at the mean rate observed over the 2004–2014 period). Shown are results for simulations assuming either 2012 (base meteorology), or 2002 (relatively warm) or 2006 (relatively cold) meteorological conditions in the future. (**e**–**h**) Corresponding monthly mean column ozone decreases south of −60° latitude.

Although the severity of ozone loss over Antarctica is most strongly affected by the abundance of reactive halogens, inter-annual variability in temperature and dynamical influences that determine strength of the polar vortex provide additional influence. For example, relatively large levels of springtime (September–November) ozone have been observed (that is, less loss) following stratospheric warming events, such as in 2002 (ref. 28), during which the occurrence of polar stratospheric clouds was relatively low^29^. The sensitivity of the SH column ozone decrease due to CH_2_Cl_2_ to a range of assumed stratospheric meteorology in the future period (see Methods) is shown in Fig. 5. In all cases, the impact of CH_2_Cl_2_ is substantial, with the greatest ozone decreases towards the end of the century predicted under the assumed 2012 (base meteorology) and 2006 (relatively cold Antarctic winter) conditions. However, note, these CTM simulations did not explicitly consider climate-driven cooling of the upper stratosphere or changes in stratospheric dynamics (see below).

## Discussion

The Montreal Protocol has been extremely successful in alleviating polar ozone loss. For example, it has been estimated the Antarctic Ozone Hole would have been 40% larger by 2013 had the Protocol not come into effect^30^. Based on current understanding, the Ozone Hole is expected to recover in this century. The 17 chemistry-climate models (CCMs) that took part in the Stratospheric Processes and their Role in Climate (SPARC) CCMVal project predict that the Antarctic Ozone Hole—defined by the October average column ozone abundance—will return to pre-1980 levels between 2046 and 2057 (ref. 7), in line with the anticipated decline in controlled ozone-depleting substances. More recent model simulations have suggested a later ozone return date that approaches the end of the century^31^,^32^, though none of these previous estimates have considered CH_2_Cl_2_, or indeed CH_2_Cl_2_ growth.

**Figure 6:**
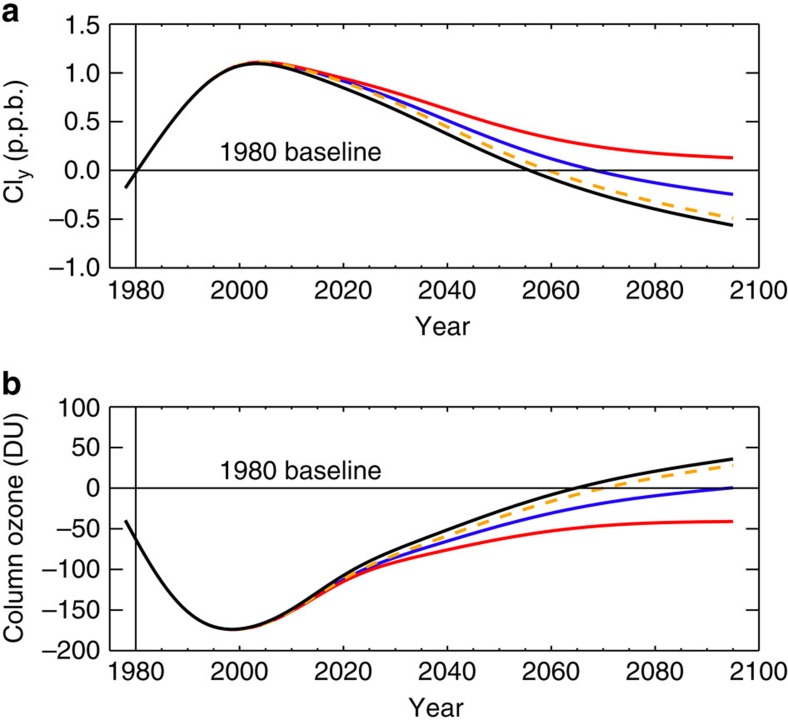
Long-term changes in stratospheric inorganic chlorine and column ozone over Antarctica. (**a**) Modelled October mean change in inorganic chlorine (Cl_y_) in the lower stratosphere (50 hPa) over Antarctica (60–90° S). Cl_y_ is expressed in parts per billion (p.p.b.) relative to a 1980 baseline. (**b**) Corresponding modelled October mean change in Antarctic stratospheric column ozone (DU) relative to 1980. Cl_y_ and ozone changes shown for SLIMCAT model simulations without CH_2_Cl_2_ (black) and for CH_2_Cl_2_ Scenario 1 (blue, surface CH_2_Cl_2_ continues to increase at the mean rate observed over the 2004–2014 period), Scenario 2 (red, surface CH_2_Cl_2_ continues to increase at the mean rate observed over the 2012–2014 period) and Scenario 3 (orange, no future growth). Note, 1980 baseline is calculated from a model simulation performed with 2012 meteorology, in a similar manner to the forward simulations, to isolate the impact of CH_2_Cl_2_ growth from inter-annual variability due to meteorology. For Cl_y_, the calculated return dates with respect to this baseline are 2056 (no CH_2_Cl_2_), 2069 (Scenario 1) and 2060 (Scenario 3). Similarly for ozone, 2065 (no CH_2_Cl_2_), 2095 (Scenario 1) and 2071 (Scenario 3).

Figure 6 shows our model estimate of Antarctic Cl_y_ and column ozone evolution with and without CH_2_Cl_2_. Antarctic column ozone returns to pre-1980 levels in 2065 when CH_2_Cl_2_ is not considered. The inclusion of CH_2_Cl_2_ delays the ozone return by 30 years under growth Scenario 1 (note, under Scenario 2 ozone does not return to pre-1980 levels this century). This is the difference that would be expected between an atmosphere without any CH_2_Cl_2_, for example, if its production was completely phased out, to an atmosphere in which sustained future growth of CH_2_Cl_2_ continues. Recall, Scenario 1 simply assumes CH_2_Cl_2_ continues to increase in the atmosphere following the average upward trend observed over the last decade. We estimate that even if the stratospheric input of CH_2_Cl_2_ remains at 2016 levels (that is, Scenario 3, a no future growth scenario), the return of ozone in the Antarctic Ozone Hole region is still delayed by ∼5 years, relative to the no CH_2_Cl_2_ simulation.

The above return dates are calculated from a 1980 ozone baseline in an atmosphere that is assumed to contain no CH_2_Cl_2_. Emissions of CH_2_Cl_2_ in 1980 are highly uncertain, owing to a paucity of atmospheric measurements at that time. However, a global emission source of ∼800 Gg CH_2_Cl_2_ per year in the early 1980s has been derived from bottom–up methods^33^. Using this estimate and adjusting the ozone baseline to account for the presence of CH_2_Cl_2_ would change the 1980 return dates to 2063 (no CH_2_Cl_2_ simulation) and 2090 (growth Scenario 1). Clearly, additional increases in atmospheric CH_2_Cl_2_ could lead to an underestimate of the timescale for stratospheric ozone recovery in coming decades. Furthermore, as CH_2_Cl_2_ already has a non-negligible impact on polar ozone, which has increased in the recent past (Supplementary Fig. 2), in the nearer term CH_2_Cl_2_ growth could confound the search for ozone recovery attributable to the phase-out of controlled ozone-depleting compounds. We note that as the Antarctic column ozone changes scale to a good approximation linearly with the Cl_y_ load under each CH_2_Cl_2_ scenario (Supplementary Fig. 3), our results can be used to estimate the impact of different future trajectories of CH_2_Cl_2_, or indeed other chlorinated VSLS, on ozone.

While the future trajectory of CH_2_Cl_2_ is uncertain, in the absence of policy controls on its production, it is likely that future trends in this gas will fall within the range of the three scenarios presented here. From Fig. 5, the absolute impact of CH_2_Cl_2_ on SH polar ozone will exhibit some year-to-year variability, depending on stratospheric meteorology (Fig. 5), though the 1980 Antarctic ozone return date delay due to CH_2_Cl_2_ exhibited a weak sensitivity to the choice of future meteorology in our SLIMCAT simulations. However, we note these runs did not consider inter-annual dynamical variability in the future or the effects of climate change, such as an acceleration of the Brewer-Dobson circulation or prolonged stratospheric cooling, as predicted by most CCMs. Although ozone trends in the Antarctic lower stratosphere are most strongly affected by trends in ozone-depleting gases (that is, the time evolution of the stratospheric halogen content) and relative to the Arctic, are less sensitive to anticipated greenhouse gas changes in the future^7^, climate change is expected to accelerate ozone recovery in this region^12^. Furthermore, given the current spread of return dates predicted by CCMs, the delay in the ozone return due to CH_2_Cl_2_ is likely to vary between models, even when considering the same CH_2_Cl_2_ growth scenarios. For a given 1980 ozone baseline, models with an inherent slower rate of ozone return will see a greater impact of CH_2_Cl_2_ on the ozone return date. This is due to the divergence of stratospheric Cl_y_ concentrations in time between an atmosphere without CH_2_Cl_2_ in the future, and one in which sustained CH_2_Cl_2_ growth occurs.

**Figure 7:**
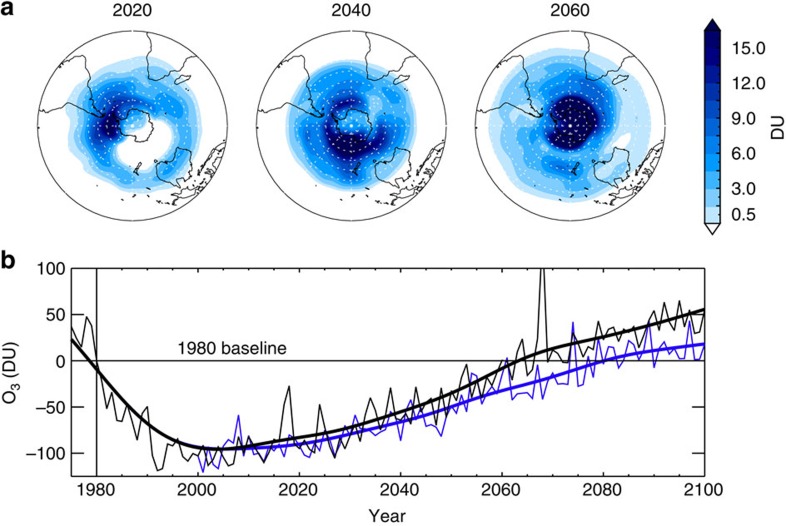
Future impact of CH_2_Cl_2_ growth on Antarctic column ozone and ozone trend from CCM simulations. (**a**) Springtime mean column ozone decrease (DU) due to CH_2_Cl_2_ in the SH. (**b**) October mean Antarctic stratospheric ozone column (DU) relative to 1980. Results are shown for UMSLIMCAT run with CH_2_Cl_2_ growth Scenario 1 (blue, surface CH_2_Cl_2_ continues to increase at the mean rate observed over the 2004–2014 period) and without CH_2_Cl_2_ (black). While inter-annual variability is large, the two ozone time series are statistically different at the 95% significance level according to a Student’s *t*-test (*P* value=0.02). Ozone returns to the 1980 baseline in the year 2064 (without CH_2_Cl_2_) and in 2081 (Scenario 1).

To explore the above, we performed simulations with the UMSLIMCAT CCM, forced by the Intergovernmental Panel on Climate Change (IPCC) RCP (Representative Concentration Pathway) 6.0 scenario, and with evolving meteorology in the future. UMSLIMCAT was evaluated in the SPARC CCMVal project^12^ and is participating in the ongoing Chemistry-Climate Model Initiative^34^ (CCMI – Methods section). Our CCM results show marked differences between a run with CH_2_Cl_2_ growth (Scenario 1) and a reference run without CH_2_Cl_2_ (Fig. 7a). The largest ozone decreases occur over Antarctica, where (i) the difference in October mean ozone between the with and without CH_2_Cl_2_ runs is statistically significant at the 95% confidence interval, and (ii) the influence of CH_2_Cl_2_ on ozone increases in coming decades—corroborating our main CTM findings. In both models, inclusion of CH_2_Cl_2_ growth delays the return of Antarctic lower stratospheric Cl_y_ to 1980 levels by 13 years. In UMSLIMCAT, column ozone return is delayed by 17 years (Fig. 7b). This is a substantial delay, albeit it is smaller than that predicted by the CTM, which assumed fixed dynamics in the future, and which predicts more severe springtime polar ozone loss (that is, for a given amount of chlorine from CH_2_Cl_2_, the ozone impact is smaller in the CCM). Ozone increases as a result of upper stratospheric cooling, a consequence of climate change^7^, may provide additional influence on the magnitude of the ozone delay from CH_2_Cl_2_ in CCM experiments. However, our CCM shows that ozone closely tracks the trajectory of Cl_y_, highlighting the dominant influence of the halogen loading on Antarctic ozone trends^7^,^12^. As previously noted, the delay from CH_2_Cl_2_ growth will vary across models and crucially these results highlight that CH_2_Cl_2_ growth should be considered within the framework of multi-model ozone assessments in the future. As the Antarctic Ozone Hole is known to affect surface climate of the SH in several ways, such as by modifying the Southern Annular Mode^35^, a delayed recovery caused by CH_2_Cl_2_ may also be relevant for refining future climate predictions.

In summary, although currently modest, the impact of CH_2_Cl_2_ on stratospheric ozone is increasing and if CH_2_Cl_2_ concentrations continue to increase they could significantly offset a portion of the decline in anthropogenic chlorine provided by the Montreal Protocol. This adds uncertainty into future assessments of ozone evolution and could lead to a significant delay in recovery of the ozone layer, particularly over Antarctica. Finally, we note that while this study has focused on CH_2_Cl_2_, for which long-term surface monitoring data exists, several other anthropogenic VSLS (for example, 1,2-Dichloroethane, C_2_H_4_Cl_2_) have been detected in Earth’s atmosphere^22^, though atmospheric measurements of these compounds are sparse. A broader consideration of the atmospheric trends of these non-Montreal Protocol compounds and other VSLS would be beneficial to improve future ozone predictions.

## Methods

### Surface CH_2_Cl_2_ measurements

Surface measurements of CH_2_Cl_2_ from NOAA’s long-term monitoring network^36^ were analysed. Results from paired flask samples collected at 13 sites were used to derive surface CH_2_Cl_2_ mixing ratios over the 2004–2014 period. The observed data were averaged in 5 latitude bins (60–90° N, 30–60° N, 0–30° N, 0–30° S and 30–90° S) and trends over the above period were derived in each bin (selected bin results are shown in Fig. 1a). These data form the basis of our current emission estimates and future CH_2_Cl_2_ growth scenarios.

### Aircraft CH_2_Cl_2_ measurements

Aircraft measurements of CH_2_Cl_2_ around the tropopause (Fig. 1d, inset) were obtained onboard the Global Hawk unmanned aircraft deployed during the 2011 and 2013 legs of the National Aeronautics and Space Administration (NASA) Airborne Tropical Tropopause Experiment (ATTREX) mission^18^. CH_2_Cl_2_ mixing ratios were derived by the University of Miami from whole air samples analysed using gas chromatography/mass spectrometry.

### Future CH_2_Cl_2_ growth scenarios

Two scenarios describing the future evolution of surface CH_2_Cl_2_ were derived by extrapolating observed surface CH_2_Cl_2_ trends in 5 latitude bins (see above). The forward scenarios cover the period 2015–2100. Scenario 1 assumes that surface CH_2_Cl_2_ increases linearly over this period, with a growth rate equal to the mean growth rate observed over the 2004–2014 period. In NH mid-latitudes, surface CH_2_Cl_2_ increases at a rate of 2.85 p.p.t. per year under Scenario 1 (Fig. 1c). Scenario 2 is a larger growth scenario and assumes that CH_2_Cl_2_ increases linearly with a rate equal to that observed over the 2012–2014 period only. In these years, CH_2_Cl_2_ growth was large compared to the decadal average used for Scenario 1, resulting in a surface trend of 6.1 p.p.t. per year in NH mid-latitudes. A third scenario, Scenario 3, was also considered in which it is assumed that no further growth of CH_2_Cl_2_ occurs beyond 2016 (that is, the surface concentration of CH_2_Cl_2_ is fixed throughout the forward simulation at 2016 levels, Fig. 1).

### Global emission estimates of CH_2_Cl_2_

A simple 1-box model, previously used to study CH_4_ and CH_3_CCl_3_ emissions, was used to derive global CH_2_Cl_2_ emissions required to sustain the observed surface concentrations. The model treated emissions of CH_2_Cl_2_ with a parameterized global mean lifetime of 0.43 years, based on this work. In the present day, global CH_2_Cl_2_ emissions estimates (Fig. 2) agree well with similar independent estimates^15^. The box model was also used to estimate that an emission source of ∼2.8 Tg CH_2_Cl_2_ per year is required to sustain the modelled 2050 atmospheric CH_2_Cl_2_ concentration under Scenario 1.

### Chemical transport model and experiment design

The TOMCAT/SLIMCAT global three-dimensional CTM^37^ was used to calculate the sensitivity of stratospheric chlorine and ozone to future growth in atmospheric CH_2_Cl_2_. The model has been widely evaluated and used in previous studies of tropospheric and stratospheric chemistry. The model is forced with meteorological fields from the European Centre for Medium-Range Weather Forecasts (ECMWF) ERA-Interim reanalysis data set. We first derived the time-dependent stratospheric injection of CH_2_Cl_2_ over the 2004–2100 period using a detailed tropospheric configuration of TOMCAT. The model contains a comprehensive description of tropospheric chemistry, including VSLS oxidation, and reproduces the tropospheric abundance of chlorine-containing VSLS well in the present day^22^. A transient simulation between 2004 and 2014 was performed in which surface CH_2_Cl_2_ was constrained in the model using the time-dependent surface measurements from NOAA, in the 5 latitude bins discussed above. Between 2015 and 2100, the model was integrated twice using the projected surface CH_2_Cl_2_ loadings from Scenario 1 and Scenario 2. We assumed present day meteorology and emissions of precursor gases that control tropospheric chemistry in the future period. The stratospheric chlorine injection from CH_2_Cl_2_ over these periods is given in Fig. 1d.

We next performed three simulations using a detailed stratospheric model configuration of TOMCAT/SLIMCAT, containing a comprehensive stratospheric chemistry scheme that includes a full description of processes relevant to polar ozone depletion. This version of the model reproduces observed stratospheric ozone trends well^21^,^30^. Experiments were performed over the 1980–2100 period at a horizontal resolution of 2.8° × 2.8° and with 32 vertical levels from the surface to ∼60 km. The stratospheric CH_2_Cl_2_ loading was prescribed in experiments Scenario 1 and Scenario 2 using the time-dependent loadings derived above. Annual averages of these data are given in Supplementary Tables 1 and 2. CH_2_Cl_2_ was only considered from 2004 onwards and the two growth scenarios diverge from 2015. A reference simulation in which CH_2_Cl_2_ was not considered (experiment No_CH_2_Cl_2_) was also performed. Time-dependent surface mixing ratios of all other long-lived ozone-depleting compounds (for example, CFCs, HCFCs, halons and others), CH_4_ and N_2_O, were taken from the World Meteorological Organization (WMO) A1 Scenario^7^,^15^. Our approach is to examine the sensitivity of future Cl_y_ and ozone to increasing CH_2_Cl_2_. Therefore, in the future period annually repeating meteorology was assumed for the arbitrarily chosen year 2012 (referred to here as ‘base meteorology’). We also examined the sensitivity of ozone changes due to CH_2_Cl_2_ growth to different and more remarkable assumed years of meteorology in the future period: 2002 (a year of relatively warm polar stratospheric temperatures) and 2006 (a year of very cold temperatures).

### Chemistry-climate model experiments

UMSLIMCAT is a global CCM that has been evaluated extensively within the recent CCMVal and CCMI multi-model inter-comparison initiatives^12^,^34^. It is based on the troposphere–stratosphere–mesosphere version of the Met Office Unified Model (UM v4.5). The model has 64 vertical levels extending from the surface to 0.01 hPa (∼80 km), and was here run at a horizontal resolution of 3.75° × 2.5° (ref. 38). The stratospheric chemistry scheme is similar to that of the SLIMCAT CTM (see above). We performed a transient control simulation without CH_2_Cl_2_ over the 1970 to 2100 period. The loading of long-lived ozone-depleting compounds and greenhouse gases was prescribed according to the IPCC RCP 6.0 scenario. A similar run but including time-varying CH_2_Cl_2_ according to growth Scenario 1 was also performed.

### Estimates of stratospheric chlorine and ozone return dates

Estimates of stratospheric Cl_y_ and ozone return dates relative to a 1980 baseline are presented. For the CTM experiments, this baseline was calculated from a 1980 model simulation run with 2012 meteorology (consistent with our forward simulations), to isolate the effect of changing composition on the return dates. Estimated Cl_y_ return date ranges are also shown (purple lines in Fig. 3) from previous CCM simulations^7^,^12^ that did not consider CH_2_Cl_2_. The model time series shown in Fig. 3 and all subsequent figures were smoothed by applying a 1:2:1 filter iteratively 30 times, consistent with previous studies^12^.

### Code availability

The TOMCAT/SLIMCAT model is supported by the Natural Environment Research Council (NERC) and the National Centre for Atmospheric Science (NCAS) and is available to UK academic institutions working with these organizations. Enquiries about the model code should be directed to M.P.C. Post processing of model output was performed using Interactive Data Language (IDL) and the code is available on request from the corresponding author (R.H.). Output from all model simulations is available on request from the corresponding author (R.H.).

### Data availability

The NOAA surface CH_2_Cl_2_ data are available to download at the following web address: http://www.esrl.noaa.gov/gmd/dv/ftpdata.html. Use of the data in a presentation, publication or report requires that users contact the principal investigator (S.A.M.) first to discuss your interests. The NASA CH_2_Cl_2_ data from the ATTREX missions are publically available at the following web address: https://espoarchive.nasa.gov/.

## Additional information

**How to cite this article:** Hossaini, R. *et al*. The increasing threat to stratospheric ozone from dichloromethane. *Nat. Commun.* **8,** 15962 doi: 10.1038/ncomms15962 (2017).

**Publisher’s note**: Springer Nature remains neutral with regard to jurisdictional claims in published maps and institutional affiliations.

## Supplementary Material

Supplementary Information

Peer Review File
