# Supplementary material for: The increasing threat to stratospheric ozone from dichloromethane
Source: Nat Commun. 2017 Jun 27;8:15962. doi: 10.1038/ncomms15962 (PMC5490265; doi:10.1038/ncomms15962)
Supplement: Supplementary Information [file ncomms15962-s1.pdf]

Type of file: PDF
Size of file: 0 KB
Title of file for HTML: Supplementary Information
Description: Supplementary Figures and Supplementary Tables

Type of file: pdf
Size of file: 0 KB
Title of file for HTML: Peer Review File
Description:

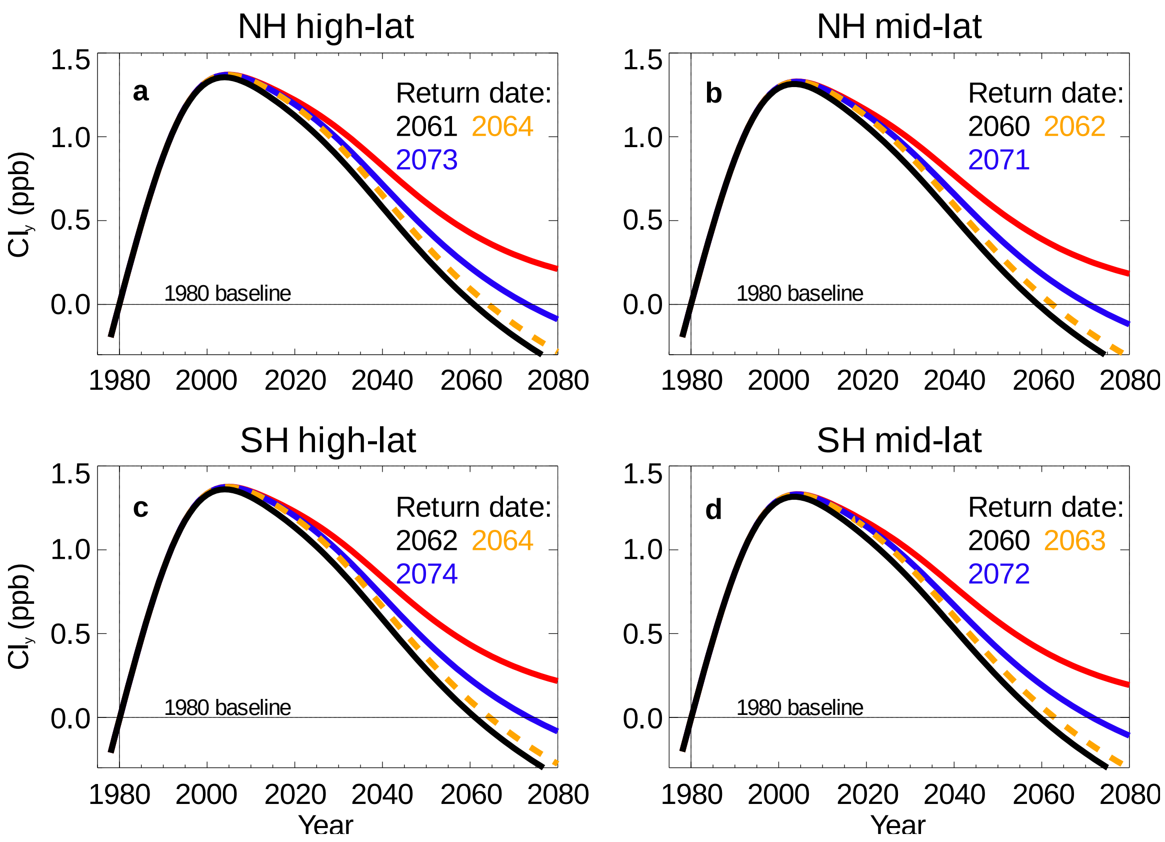

Supplementary Figure 1. Modelled inorganic chlorine (Cl_y) changes in the upper stratosphere. Cl_y changes are expressed in parts per billion (ppb) relative to 1980 baseline at 5 hPa for simulations without CH_2Cl_2 (black) and for CH_2Cl_2 growth Scenario 1 (blue) and Scenario 2 (red). **(a)** Cl_y changes in northern high latitudes (60°N-90°N). **(b)** Cl_y change in northern mid-latitudes (35°N-60°N). **(c)** Cl_y changes in southern high latitudes (60°S-90°S). **(d)** Cl_y change in southern mid-latitudes (35°S-60°S). Annotated are the projected dates when Cl_y returns to 1980 levels.

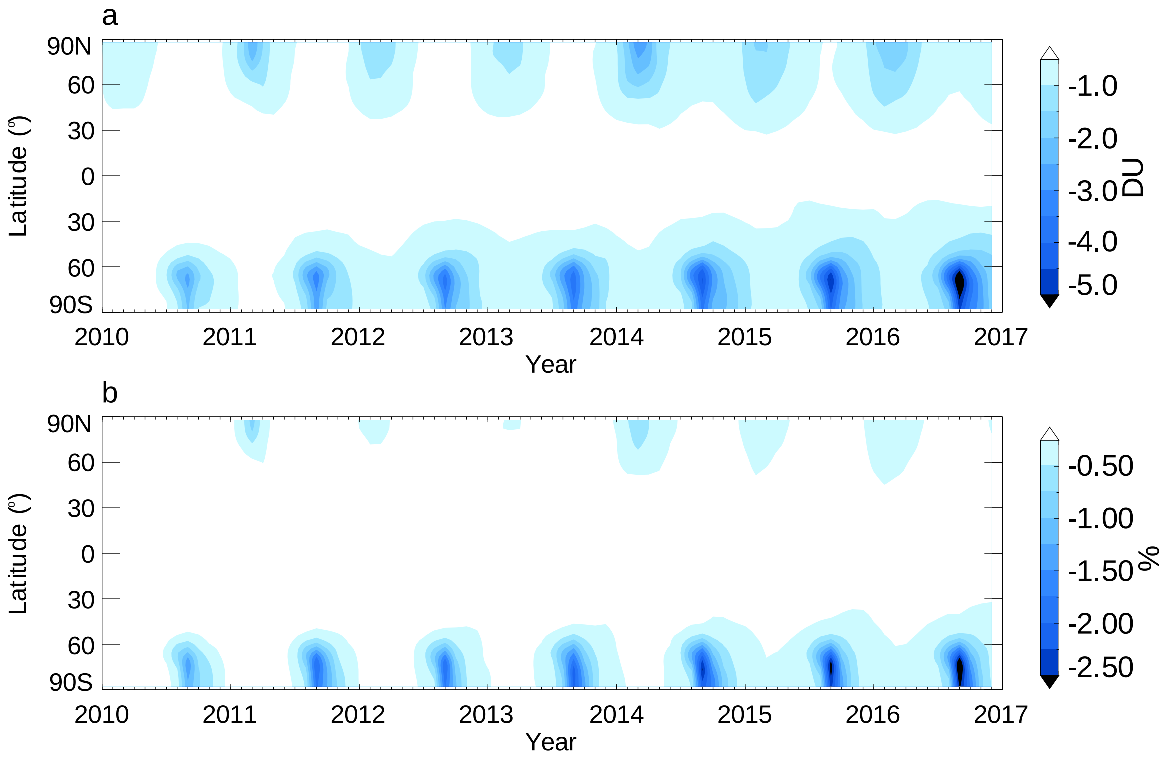

Supplementary Figure 2. Monthly stratospheric column ozone decreases due to CH_2Cl_2 in the recent past and present. **(a)** Difference in zonal mean monthly mean column ozone (Dobson units, DU) between run Scenario 1 and run no_CH_2Cl_2. **(b)** As **a** but expressed as percentage (%) change.

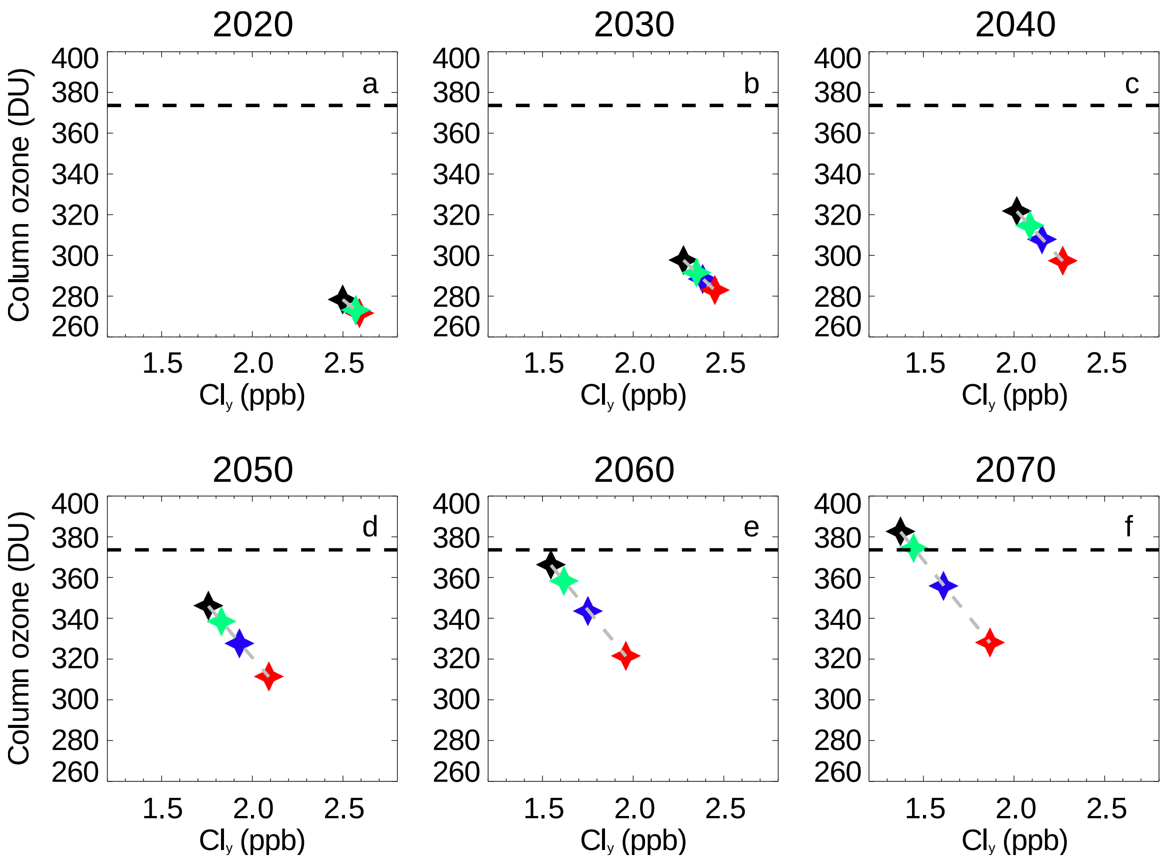

Supplementary Figure 3. Relationship between Antarctic stratospheric column ozone and inorganic chlorine (Cl_y). **(a-f)** Modelled October mean ozone column (Dobson units, DU) versus mean Cl_y (parts per billion, ppb) at 50 hPa (lower stratosphere) over Antarctica (60°S-90°S). The relationship is shown for model simulations without CH_2Cl_2 (black star) and for CH_2Cl_2 growth Scenarios 1 (blue star) and 2 (red star). The grey dashed line is a linear fit to these data. The green star represents a constant offset to the no CH_2Cl_2 case, based on 2016 stratospheric loading of CH_2Cl_2 (i.e. the ozone-Cl_y relationship with no future growth in CH_2Cl_2). The baseline column ozone amount in 1980 is shown by the horizontal dashed line.

Supplementary Table 1. Annual mean mixing ratio (ppt) of CH_2Cl_2 entering the stratosphere under Scenario 1.

Year	CH_2Cl_2	Year	CH_2Cl_2	Year	CH_2Cl_2	Year	CH_2Cl_2	Year	CH_2Cl_2	Year	CH_2Cl_2
2005	18.0	2021	44.6	2037	70.7	2053	96.8	2069	122.9	2085	149.0
2006	18.6	2022	46.2	2038	72.3	2054	98.4	2070	124.5	2086	150.6
2007	20.9	2023	47.9	2039	74.0	2055	100.1	2071	126.2	2087	152.2
2008	21.9	2024	49.5	2040	75.6	2056	101.7	2072	127.8	2088	153.9
2009	22.5	2025	51.1	2041	77.2	2057	103.3	2073	129.4	2089	155.5
2010	25.7	2026	52.8	2042	78.9	2058	104.9	2074	131.0	2090	157.1
2011	26.0	2027	54.4	2043	80.5	2059	106.6	2075	132.7	2091	158.8
2012	27.6	2028	56.0	2044	82.1	2060	108.2	2076	134.3	2092	160.4
2013	33.2	2029	57.7	2045	83.7	2061	109.8	2077	135.9	2093	162.0
2014	35.9	2030	59.3	2046	85.4	2062	111.5	2078	137.6	2094	163.7
2015	34.8	2031	60.9	2047	87.0	2063	113.1	2079	139.2	2095	165.3
2016	36.4	2032	62.5	2048	88.6	2064	114.7	2080	140.8	2096	166.9
2017	38.1	2033	64.2	2049	90.3	2065	116.4	2081	142.5	2097	168.6
2018	39.7	2034	65.8	2050	91.9	2066	118.0	2082	144.1	2098	170.2
2019	41.3	2035	67.4	2051	93.5	2067	119.6	2083	145.7	2099	171.8
2020	43.0	2036	69.1	2052	95.2	2068	121.3	2084	147.4	2100	173.4

Supplementary Table 2. As Supplementary Table 1 but for Scenario 2.

Year	CH_2Cl_2	Year	CH_2Cl_2	Year	CH_2Cl_2	Year	CH_2Cl_2	Year	CH_2Cl_2	Year	CH_2Cl_2
2005	18.0	2021	66.2	2037	130.1	2053	194.0	2069	257.9	2085	321.8
2006	18.6	2022	70.2	2038	134.1	2054	198.0	2070	261.9	2086	325.8
2007	20.9	2023	74.2	2039	138.1	2055	202.0	2071	265.9	2087	329.8
2008	21.9	2024	78.2	2040	142.1	2056	206.0	2072	269.9	2088	333.8
2009	22.5	2025	82.2	2041	146.1	2057	210.0	2073	273.9	2089	337.8
2010	25.7	2026	86.2	2042	150.1	2058	214.0	2074	277.9	2090	341.8
2011	26.0	2027	90.2	2043	154.1	2059	218.0	2075	281.9	2091	345.8
2012	27.6	2028	94.2	2044	158.1	2060	222.0	2076	285.9	2092	349.8
2013	33.2	2029	98.2	2045	162.1	2061	226.0	2077	289.9	2093	353.8
2014	35.9	2030	102.2	2046	166.1	2062	230.0	2078	293.9	2094	357.8
2015	42.2	2031	106.1	2047	170.1	2063	234.0	2079	297.9	2095	361.8
2016	46.2	2032	110.1	2048	174.0	2064	238.0	2080	301.9	2096	365.8
2017	50.2	2033	114.1	2049	178.0	2065	242.0	2081	305.8	2097	369.8
2018	54.2	2034	118.1	2050	182.0	2066	246.0	2082	309.8	2098	373.7
2019	58.2	2035	122.1	2051	186.0	2067	250.0	2083	313.8	2099	377.7
2020	62.2	2036	126.1	2052	190.0	2068	254.0	2084	317.8	2100	381.7
